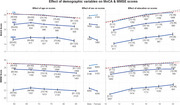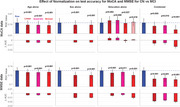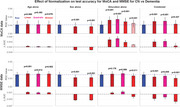# Does Demographic Normalisation Improve the Accuracy of the MoCA and the MMSE for Detecting Mild Cognitive Impairment and Early Dementia ?

**DOI:** 10.1002/alz70861_108357

**Published:** 2025-12-23

**Authors:** Kamlesh Perumal Venkatachalapathy, Maurice Smith

**Affiliations:** ^1^ Harvard University, Madurai, Tamilnadu India; ^2^ Harvard University School of Enginering and Applied Sciences, Cambridge, MA USA

## Abstract

**Background:**

The diagnosis of Mild Cognitive Impairment (MCI) is challenging and relies on accurate cognitive testing. To improve the validity of cognitive test results, demographic normalisation is commonly used to adjust test scores for the effects of factors like age, sex and education. Many studies have developed normative tables/calculators based on these demographic factors for cognitive tests widely used to identify MCI ‐ the Montreal Cognitive Assessment (MoCA) and the Mini‐Mental State Examination (MMSE). Although demographic normalisation aims to improve diagnostic accuracy, its effectiveness has been seldom studied. This is concerning because, while normalisation improves accuracy for variables unrelated to disease, the commonly adjusted demographic factors—age, sex, and education—are themselves risk factors for MCI and Alzheimer's disease.

**Method:**

We hypothesized that demographic normalisation reduces diagnostic accuracy when the demographic variables used (age, sex, education) differ systematically between diagnostic groups due to their association with disease risk. We tested this hypothesis by assessing whether normalisation affects the ability of MoCA and MMSE to distinguish MCI and early dementia from cognitively normal (CN) individuals, using receiver operating characteristic analysis with paired bootstrapping across multiple normalisation methods (bin‐based, linear, quadratic and partial).

**Result:**

Surprisingly, demographic normalisation worsened rather than improved diagnostic accuracy for detecting MCI with both MoCA and MMSE, reducing their effectiveness as diagnostic tools and confirming our hypothesis. Demographic normalisation significantly reduced diagnostic accuracy, with AUC decrements of 0.007–0.015 (2‐tailed *p* <0.001) in 7 of 8 CN vs MCI conditions, highlighting that disease‐related demographic shifts often negate normalisation benefits and lower the diagnostic performance of MoCA and MMSE. Normalisation failed to consistently improve accuracy for detecting early dementia, with significant decreases in 3 of 8 conditions and a mix of significant and non‐significant increases in the others, indicating inconsistent effects on test performance.

**Conclusion:**

The current findings indicate that normalisation with the commonly used demographic variables (age, sex, and education) has little effect on the accuracy of MoCA & MMSE for identifying MCI and early dementia. Even this little effect often results in a significantly decreased test accuracy, suggesting that demographic normalisation does more harm than good.